# Preliminary quantitative proteomics analysis in chronic and latent Keshan disease by iTRAQ labeling approach

**DOI:** 10.18632/oncotarget.22397

**Published:** 2017-11-11

**Authors:** Yuxiao Sun, Chuanyu Gao, Xianqing Wang, Yuhao Liu

**Affiliations:** ^1^ Department of Cardiology, Zhengzhou University, People's Hospital, Zhengzhou, Henan 450003, PR China; ^2^ Department of Cardiology, Henan Provincial People's Hospital, Zhengzhou, Henan 450003, PR China

**Keywords:** Keshan disease, iTRAQ, proteomics, DEPs, biomarker

## Abstract

Keshan disease is a congestive cardiomyopathy. Dietary selenium deficiency combined with additional stressors are recognized to cause the cardiomyopathies. In this study, clinical condition of individuals with different subtypes including chronic and latent were analyzed. ECG abnormalities, chest radiography, echocardiography and blood selenium concentration were assessed. Subsequently, in effort to uncover proteins that were reliably changed in patients, isobaric tags for absolute and relative quantitation technology was applied. Bioinformatics analysis of the differentially expressed proteins were performed by means of Gene Ontology classification, KEGG pathway, and Ingenuity Pathway Analysis. ELISA experiment was used to detect the interesting proteins. As a result, chronic patients showed more EGC abnormalities compared to Latent. All patients had low blood selenium level. Proteomics data revealed 28 differentially expressed proteins. By ELISA variation, LGALS3BP was increased in chronic patients. PZP was elevated specially in latent patients. The above results might be beneficial for further biomarkers discovery and Keshan disease pathological mechanism study.

## INTRODUCTION

Keshan disease (KD) is a form of endemic cardiomyopathy (ECD) that affects the heart muscle with unknown etiology. The pathological features include multifocal necrosis cardiogenic shock, arrhythmia, cardiomegaly, and replacement fibrosis of the myocardium, resulting in acute or chronic congestive heart failure. KD mainly occurs in China, involving 327 counties of 16 provinces [1]. The incidence rates are rising steadily over the past decades [2]. It's a potentially life-threatening condition and particularly widely prevalent in women and children. Clinically, KD patients are divided into four categories (acute, subacute, chronic, and latent) based on the onset of attack, clinical features, and heart function. The main subtypes are chronic and latent KD (CKD and LKD). CKD usually has insidious onset and slow progression. In terms of clinical characteristics, CKD is similar to dilated cardiomyopathy (DCM), but CKD patients have severe myocardial degeneration, evident necrosis and fibrosis, and even clear geographic features [3]. LKD is usually asymptomatic and shows mild heart enlargement with normal heart function [4].

Dietary low selenium (Se) is associated with a cardiomyopathy, myopathy and osteoarthropathy [5]. Se deficiency is considered the direct cause for KD. But how to explain the seasonal and annual variation of the disease occurrence? Moreover, not all Se deficiency regions emerge KD cases. Recent years strong evidences discuss that other factors, such as coxsackievirus B (CVB) and vitamin E (VE), contribute to KD etiology [6–8]. The incidence is likely affected by a synergistical result of various etiological factors together, including soil and water, nutrition, viral infection, geological and chemical (Se deficiency) factors. To explore the potential mechanism, molecular analysis should be performed. Quantitative proteomics have been applied to identify all the differentially expressed proteins (DEPs) in a complex mixture, which may help us understand the changes in disease oncogenesis [9]. Quantitative proteomics in heart disease disclosed that clinical outcome and risk evaluation was related to protein expression closely [10]. Recently the lectin microarray method showed the significant differences in serum glycosylation characteristics among CKD and LKD patients [11]. Thus, we think expression pattern of serum protein will give us more broad implications to KD.

**Figure 1:**
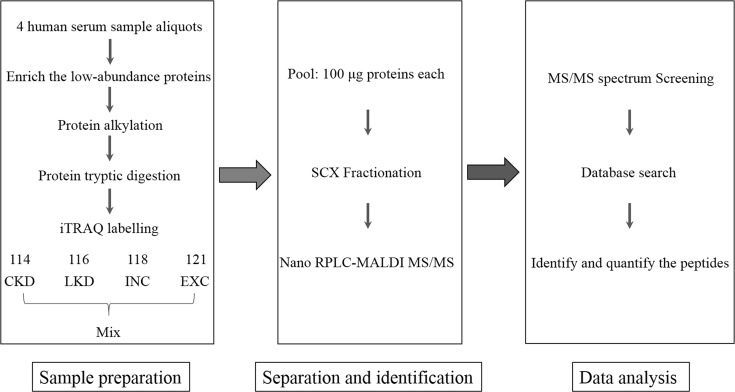
Flowchart of iTRAQ proteomics approach

Isobaric tags for absolute and relative quantitation (iTRAQ) is a mass spectrometry technology that 4 or 8 analysis samples can be quantified simultaneously in a single mass spectrometric analysis. By measuring reporter ions peak intensities of tagged peptides during MS/MS, it accurately and sensitively provides quantitative information including affinity pull-downs, time-course analyses, and elucidation of disease markers [12, 13]. Recently we have detected the CKD proteomics using iTRAQ technique and compared the protein changes with local healthy controls [14]. Aiming to expand the understand of KD, the current study collected LKD patients and controls outside the endemic areas. Epidemiological features were assessed. iTRAQ coupled with 2D-nanoHPLC-ESI-MS/MS was applied to further compare the global protein profile between CKD and LKD. The workflow of iTRAQ proteomics analysis was described in Figure 1.

## RESULTS

### Clinical baseline data of study population

The characteristics of the subjects was shown in Table 1. 31 CKD patients consisting of 17 males and 14 females with mean age 46.8 ± 9.1 years, exhibited poor heart function. The difference of age and gender had no statistical significance. Referring to the NYHA cardiac function classification method, most CKD patients belonged to NYHA class III (12, 38.7%) and class IV (12, 38.7%). 40 LKD patients consisted of 18 males and 22 females and mean age of them was 45.7 ± 11.8 years. 24 (60%) of the LKD patients showed class I with normal heart function. As the survivors experienced KD outbreak period, these patients had higher family history rate than healthy residents in affected areas.

**Table 1:** Baseline characteristics of the study population enrolled in clinical feature survey

	CKD (n=31)	LKD (n=40)	INC(n=30)
Age, yrs	46.8±9.1	45.7±11.8	44.1±13.8
Female/male	14/17	22/18	13/17
NYHA class, n (%)			
I	0(0%)	24(60.0%)	28(93.3%)
II	7(22.6%)	13(32.5%)	1(3%)
III	12(38.7%)	3(7.50%)	0(0%)
IV	12(38.76.5%)	0(0%)	0(0%)
Family history, n (%)	19(61.3%)	10(25.0%)	1(3%)

The prevalent ECG abnormalities of KD patients were shown in Table 2. Most CKD patients had several abnormalities coexisting. 3 healthy cases showed ECG abnormalities: atrial premature beat, atrioventricular block and bundle branch block. Since without conscious symptoms of cardiac insufficiency, they were classified as normal controls.

**Table 2:** Standard 12-lead ECG data of KD patients and internal controls

ECG abnormalities	CKD (n=31)		LKD (n=40)		INC (n=30)	
	Number	Ratio (%)	Number	Ratio (%)	Number	Ratio (%)
Normal ECG	1	3.2	10	25.0	27	90.0
Atrial premature beats	7	22.6	5	12.5	1	3
Ventricular premature beat(VPB)	21	67.4	6	15.0	0	0
Frequent VBP	15	48.4	3	7.5	0	0
Occasional VBP	4	12.9	3	7.5	0	0
Junctional tachycardia	1	3.2	0	0	0	0
Complete right bundle branch block	6	19.4	2	5.0	0	0
Incomplete right bundle branch block	4	12.9	3	7.5	1	3
Intraventricular block (class I)	4	12.9	4	10.0	1	3
Intraventricular block (class II)	3	9.7	0	0	0	0
ST-T changes	13	41.9	4	10.0	0	0
Ventricular hypertrophy	10	32.3	0	0	0	0
Atrial flutter	3	9.7	0	0	0	0
Qtc prolongation	4	12.9	0	0	0	0

**Figure 2:**
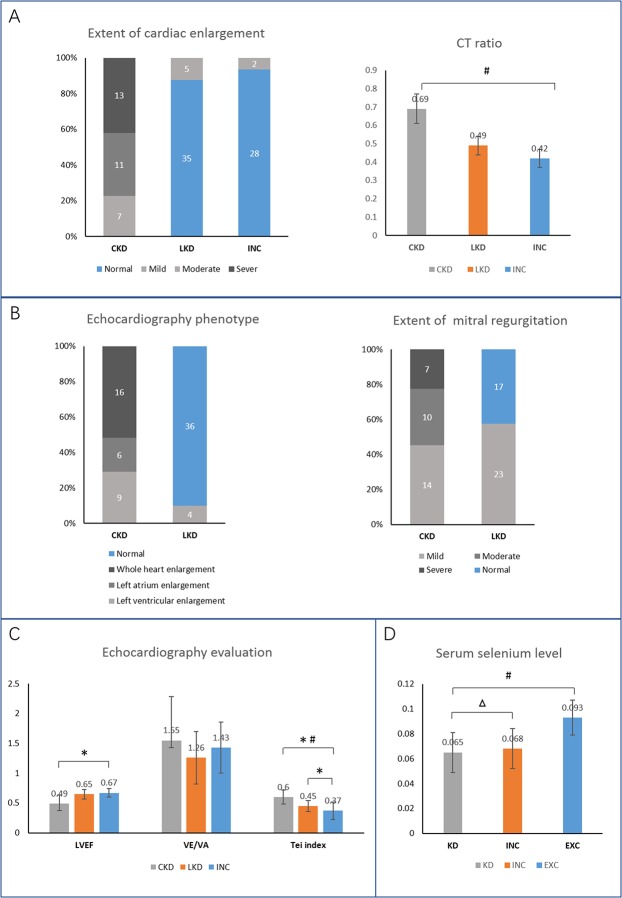
Clinical baseline data of study population **(A)** Chest radiography examination reflected the extent of cardiac enlargement. **(B, C)** Echocardiography results. **(D)** Blood selenium level in KD patients and controls at baseline. ^*^ Compared to INC, *p*<0.05; ^#^ comparison in the 3 groups, *p* <0.01; △ compared to KD, *p*>0.05.

By the chest radiography examination, 31 CKD patients were all found to have the symptom of cardiomegaly, pulmonary congestion and decreased cardiac pulsation. Majority (13, 49%) showed severe cardiac enlargement, 11 had moderate cardiac enlargement and 7 had mild. Among of the 40 LKD patients, only 5 cases showed mild cardiac enlargement. CT ratio were measured to evaluate the degree of cardiomegaly. The CT ratio of CKD group (0.69±0.08) was significantly higher than LKD (0.49±0.05) and INC group (0.42±0.05) (*p*<0.01) (Figure 2A). Echocardiography were performed. KD patients showed heart enlargement as well as mitral regurgitation. 16 CKD patients possessed whole heart enlargement and others had left atrium enlargement or left ventricular enlargement. Only 4 had left ventricular enlargement among the 40 LKD patients. 17 LKD patients had no symptom of mitral regurgitation (Figure 2B). Mitral inflow velocity in early diastole (VE) and late diastole(VA) were obtained. Left ventricular diastolic function were estimated using their ratio (VE/VA). LVEF was used to evaluate left ventricular systolic function. Meanwhile, Tei index was used to judge the systolic and diastolic function of the heart, respectively. The average values of 3 consecutive cardiac cycles were taken for all the measurements. Results showed that CKD patients had lower LVEF value and higher Tei index compared to LKD and INC. There was no significant difference in VE/VA (Figure 2C). Serum Se concentration of patients and controls were described in Figure 2D. Se level of healthy subjects in non-endemic area (0.093±0.014) were significantly higher than endemic individuals. But no statistical difference was observed between KD groups (0.065±0.016) and internal controls (0.068±0.016).

### Identification and relative quantification of DEPs

Epidemiologic feature of population participated in proteomics experiments were listed in Table 3. In the pooled serum protein sample, we identified a total of 329 proteins, of which 146 were quantified and contained at least one unique peptides (confidence interval>95%, *p*<0.05). Using EXC reporter ion m/z 121 as control, further relative quantification in each group was executed. After removing the repetitive redundant proteins, fold changes were calculated. Definitively, combined with our published data, 28 proteins had different expression profile between the four groups (Table 4). Filtered with exclusion parameters, fold changes >1.2 or <0.5 were considered significant.

**Table 3:** Baseline characteristics of the enrolled subjects in iTRAQ

Groups	CKD	LKD	INC	EXC
	N=10	N=10	N=10	N=10
Age, yrs	48.7±8.1	44.9±11.1	44.2±13.5	43.1±11.5
Female/male	6/4	5/5	5/5	5/5
Blood selenium(μg/ml)	0.065±0.017 ^*^		0.067±0.016	0.093±0.014
Family history of KD (%)	4 (40)^**^	1(10)	-	-
NYHA class, n (%)				
I	0 (0)^**^	8(80)	-	-
II	1 (10)^**^	2(20)	-	-
III	4 (20)^**^	-	-	-
IV	5 (50)^**^	-	-	-
Systolic BP(mmhg)	111±15**^#^**	117±12	120±17	123±15
Diatolic BP(mmhg)	72±9	79±7	77±9	80±8
Cardiothoracic ratio	0.63±0.06^*^	0.47±0.03	0.45±0.04	0.44±0.44
Echocardiography				
LVEF (%)	36.43±10.08^*^	56.12±10.64	64.31±8.12	67.18±7.54
V_E_/V_A_	1.32±0.29**^#^**	1.44±0.65	1.47±0.68	1.62±0.34

^#^*p*<0.05, ^*^p<0.01, ^**^ *p*<0.001.

**Table 4:** Summary of ratios, unique peptides, SC (%) and protein score of the 28 DEPs

GI_Number	Protein name	Abbreviation	Fold change						Unique peptides	SC (%)	` Protein score
			CKD/INC	CKD/EXC	LKD/INC	LKD/EXC	CKD/LKD	INC/EXC			
gi5031863	Galectin-3-binding protein	LGALS3BP	3.352241	3.725793	1.47615	1.881961	1.980342	1.347234	4	68.63	217
gi62871078	Immunoglobulin alpha heavy chain variable region	-	1.515717	1.827663	1.443929	1.283426	0.913831	-	9	12.34	162
gi10636875	Immunoglobulin heavy chain variable region	-	1.36604	1.197479	1.526259	1.319508	0..888843	-	7	28.04	218
gi31397	Fibronectin precursor	FN1	1.292353	1.180993	1.239708	1.156688	1.035265	-	46	21.56	1620
gi1620909	Ceruloplasmin	CP	1.265757	1.248331	-	-	1.057018	-	2	8.05	138
gi177870	Alpha-2-macroglobulin precursor	A2M	1.265757	1.06437	1	1	1.394744	0.806642	3	11.53	247
gi573114	C1q B-chain precursor	C1QB	1.248331	1.239708	-	-	1.049717	-	4	25.71	101
gi4502261	Antithrombin-III precursor	SERPINC1	1.148698	0.882703	1.375553	1.257013	0.697372	-	27	41.16	650
gi77744385	Complement factor H	CFH	1.148698	1.042466	2.114036	2.056228	1.526259	-	4	9.5	281
gi41388180	Monoclonal IgM antibody heavy chain	-	1.140764	1.079228	1.205808	0.888843	1.021012	-	26	30.85	615
gi115298678	Complement C3 precursor	C3	1.125058	0.933033	1.337928	1.042466	0.920188	-	99	43.48	2549
gi37138	Unnamed protein product	THBS1	1.071773	0.773782	1.180993	1.189207	0.870551	0.707107	7	12.65	375
gi182412	Coagulation factor V precursor	F5	1.049717	0.97942	1.613284	1.231144	0.806642	-	2	3.82	172
gi130675	Serum paraoxonase/arylesterase 1	PON1	1.028114	1.117287	1.148698	1.125058	1.071773	-	7	33.24	328
gi178849	Apolipoprotein E	APOE	1	0.852635	0.864537	0.882703	0.986233	-	41	52.05	735
gi33989	Inter-alpha-trypsin inhibitor heavy chain	ITIH1	1	1.197479	1	1.301342	0.920188	1.239708	8	32.13	229
gi2258128	Complement 9	C9	0.607097	0.835088	0.532185	0.721965	1.132884	1.310393	2	6.75	106
gi33985	Trypsin inhibitor	ITIH2	0.773782	1.265757	1.057018	0.852635	1.140764	1.248331	3	13	279
gi1655598	Lipopolysaccharide binding protein	LBP	0.864537	0.876606	1.079228	1.071773	0.586417	-	9	17.05	271
gi190026	Plasminogen	PLG	0.888843	1.414214	0.757858	1.180993	1.172835	1.515717	2	11.36	144
gi13477169	Vitronectin	VTN	0.895025	1.021012	1	1.156688	1.057018	-	54	26.15	449
gi42716297	Clusterin isoform 1	CLU	0.913831	0.97942	0.926588	0.972655	1.006956	-	42	27.15	458
gi35825	Pregnancy zone protein	PZP	0.913831	1.392352	1.603851	3.961702	0.659754	2.173470	2	47.89	199
gi35825	Proapolipoprotein	APOA1	0.920188	1.021012	0.933033	1	1	-	148	70.68	868
gi178775	Unnamed protein product	F2	0.965936	0.876606	0.870551	0.82932	1.125058	-	40	37.14	907
gi189066554	C4b-binding protein alpha chain precursor	C4BPA	0.972655	1.042466	1.057018	0.933033	0.965936	-	9	23.45	333
gi4502503	Alpha-1-antitrypsin	SERPINA1	0.993092	0.558644	2.20381	1.210335	0.450625	0.532185	2	34.21	240
gi4502149	Apolipoprotein A-II preproprotein	APOA2	-	1.464086	-	1.189207	-	-	9	54	174

“-” means no data

**Figure 3:**
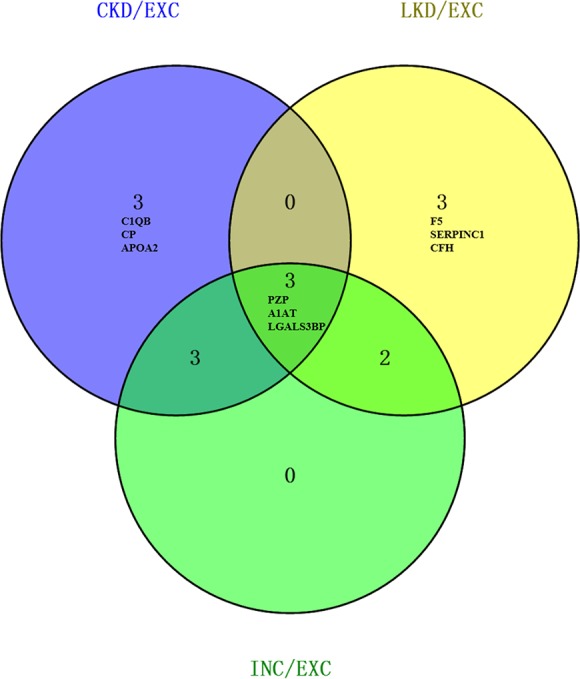
Venn diagram of significantly DEPs compared to EXC The numbers in each large circle represented the total number of proteins among various combinations; the overlap represented common proteins. Probable biomarkers within our results were exhibited.

A sharp increased level of LGALS3BP was observed in KD, especially in CKD. Individuals in endemic regions had a high PZP concentration, particularly in LKD. But compared to INC and LKD, PZP in CKD was decreased. The Venn-diagram in Figure 3 shows the overlap of significantly DEPs among CKD, LKD and INC compared to EXC. Respectively, there were 3 proteins (C1QB, CP, APOA2) remarkably altered in CKD and 3 (F5, SERPINC1, CFH) in LKD.

### Bioinformatics analysis of DEPs

**Figure 4:**
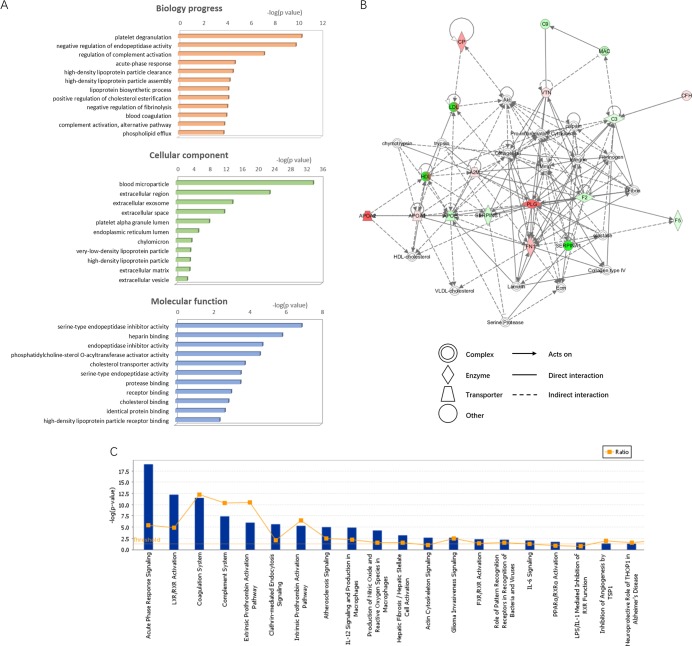
Bioinformation of the DEPs were shown **(A)** The identified proteins were divided into 3 categories: cellular component, molecular function and biological process. The top components 10 (assigned by *p* value) are presented here. **(B)** IPA network analysis (ID 1, score 34). Take CKD vs EXC as an example. Red, up-regulated proteins; green, down-regulated proteins; white, proteins are involved in certain network but not identified in this study. The degree of alter for proteins is displayed by color depth. **(C)** IPA pathway analysis. -log (*p* value) of terms ranked 1-20 were showed. Vertical axis in right means the protein ratios.

By Go functional analysis, the 28 proteins were divided into 3 categories using DAVID v6.7: molecular functions, biological processes and cellular component. In Figure 4A, for biology progress, the main terms were platelet degranulation (*p*=4.20E-11), negative regulation of endopeptidase activity (*p*=1.30E-10) and regulation of complement activation (*p*= 5.90E-08). For cellular component, majority of the proteins were involved in blood microparticle (83%), extracellular region (100%), extracellular exosome (91%). In respect to molecular function, they were associated with serine-type endopeptidase inhibitor activity (*p*=1.10E-07), heparin binding (*p*=1.30E-06) and endopeptidase inhibitor activity (*p*=1.60E-05). Pathway enrichment analysis showed that 28 DEPs participated in 8 pathways. Among them, Complement and coagulation cascades (ko04610) covered 11 proteins (A2M, F2, F5, C1QB, C3, C9, C4BPA, CFH, PLG, SERPINA and SERPINC). The networks analysis and signaling pathway annotations were computed by IPA tools. These proteins were shown to be involved in 5 key protein-protein interaction networks ranged between 34 and 2 (Table 5 and Figure 4B). The primarily clustered network (score 34) containing 15 focus molecules (A2M, APOA1, APOA2, APOE, C3, C9, CFH, CP, F2, F5, FH1, PLG, SERPINA1, SERPINC1 and VTN) was displayed in Figure 4B. Pathway category was displayed in Figure 4C. Acute phase response signaling, LXR/RXR activation, coagulation system and complement system, and Extrinsic prothrombin activation pathway were the top 5 pathway annotations.

**Table 5:** IPA Networks ranking with relative molecules symbols

ID	Molecules in network	Score	Focus molecules	Top functions
1	A2M, Akt, APOA2, APOE, C3, C9, calpain, CFH, chymotrypsin, Collagen type IV, Collagen (s), CP, Ecm, elastase, F2, F5, Fibrin, Fibrinogen, FN1, HDL, HDL-cholesterol, Intergrin, Laminin, LDL, MAC, Map, Pld, PLG, Pro-inflammatory Cytokine, Serine Protease, SERPINA1, SERPINC1, trypsin, VLDL-cholesterol, VTN	34	15	Cellular Movement, Hematological System Development and Function, Immune Cell Trafficking
2	BRAF, chemokine, CPL7A1, DHCR24, ERK1/2, gelatinase, HRG, IgG, IL1, IL12 (complex), IL12 (family), ILR1, Immunoglobulin, indicant, Jnk, LBP, LGALS3BP, Mapk, MIR320, MMP10, NFkB (complex), P38MARK, Pak, Pdgf (complex), PI3K (complex), Pka, Pld, Rac, SERPINC1, SERPINF2, Tgf beta, THBS1, TLR2/TLR4, Vegf	5	3	Cancer, Endocrine System Disorders, Carbohydrate Metabolism
3	Iti, ITIH1, ITIH2, ITIH3, ITIH4	5	2	Cardiovascular Disease, Hereditary Disorder, Metabolic Disease
4	APOA1, APOE, CD47, CD59, cd59a, CTSD, cyclic GMP, dihydrotestosterone, EDN1, F2, Hba, HBB, Hbb-b2, heme, hemoglobin, HIF1A, HMOX1, homocysteine thiolactone, HP, IL10RB, iron, ITGB3, JAK2, lipid, MIF, NOS3, NR2C2, PDGFB, SCARB1, SLC40A1, SMAD7, SNAI2, SOD1, SPP1, TNFRSF9	5	3	Hematological System Development and Function, Hematopoiesis, Tissue Morphology
5	C1q, C1QB, DMP1, DYSF, PPARA, PPARD, PSEN1, PSEN2	2	1	Organ Morphlogy, Hair and Skin Development and Function, Hereditary Disorder

The statistical likelihood (score) was used to rank the networks. Score value, number of molecules and top function for each network were listed

### ELISA detection

**Figure 5:**
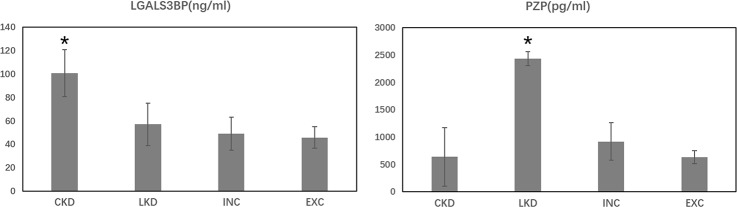
Protein serum concentration of LGAL3BP and PZP in the four groups ^*^p<0.01 vs other groups.

Galectin-3 binding protein (LGALS3BP) and Pregnancy zone protein (PZP) were selected for quantitative proteomic analysis verification using ELISA (n = 20). Differences between the four groups (*p* < 0.05) were noted. The results were consistent with iTRAQ in Figure 5. Expression level of LGALS3BP in CKD (100.72±19.97 ng/ml) was up-regulated significantly when compared with LKD, INC and EXC. While the expression of PZP was significantly increased in LKD (2439.15±533.67 pg/m) compared with CKD, INC and EXC (*p* <0.05).

## DISCUSSION

Clinical data provided important reference and guidance for KD diagnose. CKD remains a higher mortality and always developed from untreated LKD patients [15]. Almost all CKD cases exhibit severe abnormal ECG changes in present study. For KD inspection, ECG measurement is a common method. Besides, ECG has high sensitivity for CKD risk prediction in LKD. Previous studies have demonstrated that the presence of major ECG abnormalities among LKD was associated with a higher risk of CKD [4]. In our investigation, junctional tachycardia, complete bundle branch block, intraventricular block, ventricular hypertrophy, and atrial flutter were considered as major abnormalities. The criteria for minor prevalent ECG abnormalities were premature beats and incomplete bundle branch block. The 41 LKD patients mainly showed atrial premature beats, ventricular premature beat (VPB), incomplete right bundle branch block and ST-T changes. Several cases showed complete right bundle branch block and intraventricular block (class I). X-chest radiography and echocardiography provides important application value for KD progresses. Severe cardiac enlargement, obvious pulmonary congestion, decreased apex beat and a significantly increased CT ratio were found in CKD patients. But in terms of the imagological examination, CKD is not obvious difference from DCM symptoms. Epidemiological characteristics including extent of valvular regurgitation and cardiac function index are of great value for KD identification from other myocardial disorders.

CKD patients suffered from severe myocardial degeneration, necrosis and fibrosis. Obviously decreased LVEF of the 31 CKD patients indicated that their heart systolic function was damaged, which may increase the heart preload to maintain the effective stroke volume via Starling's law. Long-term heart injury exacerbates cardiac chambers enlargement and cardiac compensatory decompensation. Elevated Tei value reflects the impaired left ventricle. For LKD, the heart cavity is slightly enlarged. 57.5% patients have mild mitral regurgitation. There were no evident difference on LVEF and VE/VA compared to CKD and normal group, but the Tei value is significantly raised. The result hinted that slight damage appeared during compensated stage in LKD patients. Tei index is more sensitivity to detect mild cardiac dysfunction in early stage of LKD.

KD patients participated in our investigation presented low blood Se. Se-deficiency induced decreased activities of selenium-dependent antioxidant enzymes such as GPx1 and TrxR1 [16, 17]. Excessive oxidative stress was positively correlated with the degree of myocardium damage. Although epidemiologic studies have assessed the prevalence of KD and chronic exposure to environmental Se [18, 19], alternative etiologic hypotheses discussed it's possibly being a cofactor or simply an innocent bystander. This is what we need to think deeply. Notwithstanding LKD have a high prevalence rate, since hidden onset and asymptomatic character, the surveillance and diagnosis usually needs a long-term follow-up and clinical monitoring [20]. Traditional methods such as ECG, X-ray and echocardiography are not enough to predict the exacerbation. Therefore, prevention efforts could focus on the KD pathogenesis. Proteomic studies of KD have been conducted by two-dimensional gel electrophoresis, ClinProt Technique and microarrays [21–23]. Given meaningful proteins are masked by the high-abundant proteins (HAPs) in serum, LAPs detection is pivotal. ProteoMiner Protein Enrichment protocol is an effective strategy to capture serum LAPs [24]. In addition, the iTRAQ shot-gun approach offer us a better option with higher sensitivity.

Expression profile of the 28 identified proteins changed apparently in KD subtypes. Basing on KEGG database, 47.8% of the proteins were linked to the coagulation and complement cascades. The cascades appear to be triggered simultaneously by severe tissue injury, acute trauma, or during systemic inflammation [25]. We suggested that its activity was led by the damaged myocardial cells.

Moreover, increasing levels of complements, CP and C1QB were seen only in patients with CKD. CP had ability to evaluate the activity of vascular inflammation and pathophysiological processes of heart failure (HF) [26, 27]. C1QB is discovered as part of C1 and participates in a variety of cellular processes independent of complement activation, such as innate and adaptive immunity and wound repair [28]. The C1QB functions in impaired myocardium may be worth studying. Elevated serum level of LGALS3BP was detected in both CKD and LKD. Especially in CKD, who suffer from more severe myocardial fibrosis, the rising fold changes reached to 3.352241 and 3.725793 versus INC and EXC respectively. Compared to LKD, LGALS3BP in CKD was also significantly raised (1.980342). LGALS3BP was frequently reported to be a critical inflammatory mediator for anti-virus and anti-bacteria immune responses [29]. Recently studies suggested LGALS3BP involved in potentiate monocyte-derived fibrocyte differentiation and late fibrosis was characterized by an increased LGALS3BP [30]. Combined with the clinical characteristics, the current work indicate LGALS3BP may be a predictor assessing CKD fibrosis severity. But the result needs further study on a large-scale sample size to confirm.

SERPINC1 and F5 increased in LKD, but in CKD, they decreased. SERPINC1 is the most important coagulation factor inhibitor and suppress inflammation. SERPINC1 deficiency caused Thromboembolic. Numerous studies proved that reduced SERPINC1 have a superiority in predicting many disorders (e.g. heart failure, kidney diseases) [31, 32]. Upregulation of PZP in LKD was first revealed in present study. PZP and its homologous glycoprotein A2M were also considered inhibitors of coagulation [33]. High SERPINC1 and PZP level reflects LKD patients may have a lower risk of vascular embolization. Unlike SERPINC1, PZP is quantitatively an important pregnancy-associated protein and strongly influenced by hormones. But we guaranteed that there were no participates during pregnancy or under hormonal treatment. It is an interesting discovery. Svendsen P *et al* found that PZP intravenous significantly prolonged the survival of heterotopic A/J heart transplants in A.CA mouse recipients. [34] In 2016, John D. Eicher *et al* conducted RNA-seq studies on 9,565 platelets transcripts from 32 acute myocardial infarction (MI) patients. The data displayed higher PZP expression in NSTEMI platelets than STEMI [35]. Beyond that, PZP was rarely reported to have correlation with any myocardial diseases. Based on fact that PZP has been found elevated in numerous inflammatory states and evidence that elevated PZP eventually evoke the collagen reactivity [36], we present PZP exerts an unspecific immunosuppressive effect in dysregulated cardiomyocytes. Since the low degree of myocardial fibrosis in LKD, we deem PZP may delayed the appearance of corresponding symptoms in some extent. However, perennial inflammation progress in CKD may influence PZP expression. This hypothesis needs to be further demonstrated.

In summary, clinical characteristics and KD proteomics in chronic and latent subtypes were investigated. The present study is the first to compare the proteome in CKD and LKD using iTRAQ method. We found LGALS3BP increased distinctly in CKD. PZP increased in LKD but decreased in CKD. ELISA test certified it. Our findings contribute a prospective insight to KD molecular mechanism.

### LIMITATIONS

Because of some restriction in our research, there are limitations and deficiency to the present study. First, the serum sample size for proteomic analysis is small, large-scale of patient samples await a long-term collection and perform. Involvements of acute or subacute KD cases would provide more enlightenment. Then, the related signaling pathways and potential biomarker needs more samples and clinical experiments to confirm.

## MATERIALS AND METHODS

### Study population

A total of 131 participates were recruited for the clinical characteristics investigation. 31 CKD patients and 40 LKD patients from severe KD endemic areas Yunyi County and Huangling County (Shaanxi, China) were filtered with strict clinical criterion in new “National Criteria for Diagnosis of the Keshan Disease” (WS/T210-2011) promulgated by National Ministry of Health. The patients were newly or past diagnosed as Keshan disease with a long-term follow-up. 30 internal volunteers (INC) from KD endemic areas and 30 external volunteers (EXC) presented at Physical Examination Department of the second affiliated hospital of Xi'an Jiaotong University (Shaanxi, China) were collected as healthy controls. They matched with KD patients for age and gender (detailed data were presented in Table 1). For iTRAQ based quantitative proteomics analysis, blood samples from 20 KD patients including 10 CKD and 10 LKD, 20 healthy controls including 10 INC and 10 EXC were obtained.

Ethics approval was granted by the ethics committee of the second affiliated hospital of Xi'an Jiaotong University. Signed informed consent was obtained from all participants (or their legal guardians). KD patients and INC individuals must have lived in the KD-affected areas for consecutive period of at least six months. Individuals were excluded if they met any of the criteria described below:

- dilated cardiomyopathy patients;
- bone metabolic disease patients and kidney disease patients;
- high blood pressure, coronary heart disease, diabetic pulmonary fibrosis;
- being pregnant or lactating;
- other chronic diseases.

### Clinical evaluation

Physical examination, standard 12-lead electrocardiography (ECGs, ECG-1250C/P, JAPAN), chest radiography (Simens, Germany) and echocardiography (Paker SonoNet-3D, USA) were performed at baseline in KD patients and internal healthy subjects strictly using standardized procedures. Cardiac functions of all the endemic area subjects were classified referring to the NYHA (New York heart association) classification method. The average values of 3 consecutive cardiac cycles were taken for all the measurements.

Specifically, the criteria for prevalent ECG abnormalities in KD patients were any of the following: premature beats, junctional tachycardia, (left and right) bundle branch block, intraventricular block, ST-T changes, ventricular hypertrophy, atrial flutter, and QTc prolongation. Participants with no ECG abnormalities were classified as normal ECG. Cardiothoracic ratio (CT ratio) was measured to determine the extent of cardiac enlargement. Normal CT ratio ≤0.50. Cases with CT ratio >0.50 were diagnosed as cardiac enlargement: 0.51-0.55 were classified as mild enlargement, 0.56-0.60 were classified as moderate enlargement, >0.60 were classified as severe enlargement. As for echocardiography evaluation, mitral inflow velocity in early diastole (VE) and late diastole (VA) were obtained. Left ventricular diastolic function were estimated using their ratio (VE/VA) and left ventricular systolic function was evaluated by left ventricular ejection fraction (LVEF). Tei is a comprehensive index of reflecting cardiac systolic and diastolic functions. 5ml fasting venous blood samples of all the participates were collected and serum was obtained through centrifugation at 4,000 rpm for 10 min and stored at −80°C. Serum selenium concentrations were measured using a fluorescence spectrophotometric method (XDY-2A; AODI Detection Instrument CO., Beijing, China).

### Protein preparation, iTRAQ labeling and SCX fractionation

According to the manufacturer's instructions, serum samples of the 40 subjects were processed using ProteoMiner^TM^ Protein Enrichment Systems (Bio-Rad, USA) to reduce the complexity and enrich the low-abundance proteins (LAPs). Protein samples were denatured using 20ul dissolution buffer and 1ul 2% SDS, reduced using 10mM DTT at 56°C for 1h, alkylated using 55mMIAM in darkness for 1h, and then precipitated within chilled acetone (4×volume) for 2h. After being centrifuged at 20,000g for 30min, the pellet was next dissolved in 1%SDS and 50% TEAB (9×volume). Protein concentrations were determined using 2D Quant Kits (GE Healthcare, Buckinghamshire, UK). Removal efficiency of high-abundance proteins was detection by SDS-PAGE gel. Samples were digested using Trypsin (sigma, USA) at 37°C for 24h (protein: trypsin = 25:1). Digested peptides were lyophilized to complete dryness and stored at −80°C until needed.

ITRAQ labeling was carried out with an iTRAQ® Reagent-8Plex Multiplex Kit. (Applied Biosystem, Foster City, CA) according to manufacturer's protocol. Each lyophilized sample (100ug) was dissolved in 0.5M TEAB and added with 70ul isopropyl alcohol. Peptides were labeled with respective isobaric tags (114 for CKD; 116 for LKD; 118 for INC; 121 for EXC), incubated for 2 h, and blend. 10×volume buffer A (10mM KH2PO4, 25% ACN, pH3.0) was mixed to labeled peptides. The pooled sample was then fractionated using Phenomenex Luna 5u SCX 100A (250×4.6 mm, 5micron, Phenomenex, USA) at a flow rate of 1.0 mL /min with Buffer A and Buffer B (Buffer A with 2M KCl). SCX Fractionation was followed by a C-18 reversed phase (RP) nano-column (100μm,75um, 5micron, Micron Technology, Boise City, USA) elution using an Proxeon Easy nano-LC system (Proxeon, Thermo, USA) at a flow rate of 300 nL/min with buffer A (95%water, 95% acetonitrile,0.1%formic acid) and buffer B (95% acetonitrile, 5%water,0.1%formic acid). A continuous linear binary acetonitrile gradient was applied: 5% Buffer 8in 0-80min; 45% buffer B in 80-85min; 80% buffer B in 85-105min; 5% buffer B in 105-120min, stop. Ultimately, 17 tubes of fractions separated from the column were vacuum concentrated.

### ESI-MS/MS proteomic analysis and data screening

The peptides were subjected to nano-electrospray ionization followed by tandem mass spectrometry (MS/MS) in a Q-TOF coupled online to the HPLC system. The 3 most intense precursors from MS1 spectra were applied for MS2. Survey scans were acquired from m/z 50 to 3000. The original MS/MS file data were collected with micro TOF-Q control (Bruker, Germany) and submitted to DataAnalysis4.0, from which raw data was converted to MGF file. The Mascot search software (Matrix Science, London, UK) were used to identify and quantify the peptides. The Mascot search parameters were as follows: Enzyme: trypsin; Database: NCBInr_human; Peptide Charge:(1+, 2+ and 3+); Instrument: ESI-QUAD-TOF; Mass values: monoisotopic; Peptide mass tolerance: ±1Da; Fragment mass tolerance: ±1Da; Sample Type: iTRAQ 8plex (Peptide Labeled); Data format: Mascot generic; Fixed modification: carboxymethyl (C); Variable modifications: Gln->pyro-Glu(N-term Q), Oxidation(M), iTRAQ8plex(K), iTRAQ8plex(Y), iTRAQ8plex (N-term).

To reduce the probability of false peptide identification, only peptides with significance scores (≥ 40) at the 95% confidence interval were counted as identified. At least one unique peptide was involved in each confidently identified protein. The fold change cutoff ratio < 0.8 or >1.2 was determined to designate differential expression proteins (*p* < 0.05) [37].

### Bioinformatics analysis

Identified proteins were imported the online software DAVID (https://david.ncifcrf.gov/) to perform gene ontology (GO) analysis. GO terms were annotated on the following three aspects: molecular function, cellular component, and biological process. Pathway enrichment based on KEGG pathway database uncovered the enriched pathways of altered proteins. Additionally, the biological network and pathway analysis of these proteins were performed using Ingenuity Pathway Analysis software (IPA, www.ingenuity.com), which is based on the published literatures.

### Protein expression verification by ELISA

To verify the iTRAQ results, the serum levels of selected proteins were detected using an ELISA quantitation kit (Cusabio, Wuhan, China) following protocols recommended by the manufacturer. Triplicate samples were measured in random order.

### Statistical analysis

Continuous variables are described by means ± SD. SPSS13.0 software (IBM, Armonk, NY, USA) was used for statistical treatment of clinical data. Comparisons between groups were performed using χ2 test for categorical variables and Student's t-test for continuous variables. Two-sided *p* value<0.05 was statistically significant.
